# Estrategias para la utilización de la tomografía de haz cónico en dientes con sospecha de fractura radicular. Reporte de serie casos

**DOI:** 10.21142/2523-2754-1004-2022-136

**Published:** 2023-12-26

**Authors:** Rosanna Landa de Bellera

**Affiliations:** 1 División de Endodoncia, Cidem Imagenología. Valencia, Carabobo, Venezuela. rosannabellera@gmail.com División de Endodoncia Cidem Imagenología Valencia, Carabobo Venezuela rosannabellera@gmail.com

**Keywords:** tomografía computarizada de haz cónico, fisuras dentales, endodoncia, cone-beam computed tomography, dental fissures, endodontics

## Abstract

Las fracturas radiculares constituyen, después de la caries y la enfermedad periodontal, la tercera causa de pérdida de piezas dentarias, y son muy comunes en dientes posteriores y endodónticamente tratados. La sintomatología que producen es ambigua y su diagnóstico depende del engranaje de múltiples factores clínicos e imagenológicos. Entre los estudios diagnósticos disponibles para establecer el origen de los síntomas está la tomografía computarizada de haz cónico (TCHC). El objetivo del presente reporte de casos es la descripción de las estrategias empleadas en la adquisición e interpretación de la TCHC en tres pacientes con la sospecha de fracturas radiculares. El uso de un campo de visión reducido, con un voxel de 0,150 mm o menor, acompañado por la exploración dinámica del volumen, posibilitaron la detección de defectos óseos como signo indirecto para la localización de las líneas de fractura radicular.

## Abstract (en)

Root fractures are after caries and periodontal disease, the third cause of loss of dental units, being very common in posterior and endodontically treated teeth. The symptoms they produce are ambiguous and their diagnosis depends on the combination of multiple clinical and imaging factors. Among the diagnostic studies available to establish the origin of the symptoms is the cone beam computed tomography (CBCT). The objective of this case report is to describe the strategies used in the acquisition and interpretation of the CBCT in three patients with suspected root fractures. The use of a reduced field of view, with a voxel of 0.150 mm or less, accompanied by dynamic volume exploration made it possible to detect bone defects as an indirect sign for locating root fracture lines.

## INTRODUCCIÓN

La Asociación Americana de Endodoncia define la fisura como una delgada disrupción del esmalte, la dentina y, posiblemente, el cemento, con profundidad y extensión desconocidas. Por su parte, la fractura muestra una mayor profundidad, lo que causa una franca disrupción de los tejidos duros del diente, visible o no, ya sea de manera clínica o radiográfica ^1^. El diente fisurado y la fractura radicular vertical están presentes longitudinalmente en la estructura dentaria de manera completa o incompleta, y no son excluyentes, debido a que la fisura puede progresar en el tiempo, por lo que se convierte en una fuente de contaminación y enfermedad pulpar y periapical. Las fisuras y fracturas dependen del diagnóstico temprano para aumentar la posibilidad de conservar el diente o preservar el hueso de soporte con la finalidad de sustituirlo por un implante ^2^.

Las fracturas radiculares constituyen después de la caries y la enfermedad periodontal la tercera causa de pérdida de unidades dentarias ^3^, son más comunes en dientes endodónticamente tratados (3,69-25%) ^(3, 4)^ y más frecuentes en dientes posteriores, entre ellos los premolares superiores y molares inferiores ^5^^,^^6^. Se considera que su frecuencia aumenta con la edad, y es mayor entre los 30 y 69 años, sin predilección por sexo ^3^.

El estudio diagnóstico para la detección de fisuras o fracturas incluye la sintomatología, el test de vitalidad pulpar, la mordida y oclusión, la transiluminación, la remoción de restauraciones, la pigmentación, el sondaje periodontal y radiografías periapicales, y es posible tener que recurrir a técnicas quirúrgicas exploratorias que la confirmen cuando su localización es radicular. La pigmentación y exposición quirúrgica representan el *gold standard* en confirmación ^4^^,^^7^.

Las radiografías periapicales (RP) son el primer recurso para el diagnóstico imagenológico, pero presentan limitaciones como la distorsión geométrica y el ruido anatómico al comprimir estructuras tridimensionales en una imagen bidimensional, lo que provee la observación del plano mesiodistal, con muy poca visualización del plano bucolingual/palatino. Pueden detectarlas solo cuando existe un desplazamiento entre los fragmentos, si este desplazamiento está en el mismo plano de la dirección de los rayos X y existe una mínima superposición ^8^. En las RP, la apariencia puede ir desde ningún cambio o sutiles pérdidas de hueso perirradicular, que quedan enmascaradas por las tablas óseas íntegras, a defectos óseos verticales como el típico defecto en forma de “J” presente en fracturas verticales de larga data, y radiolucencias que comprometen la región de furca o ubicadas en la base de un perno, que pueden observarse mesial o distal a la raíz comprometida ^6^. 

La tomografía computarizada de haz cónico (TCHC) es un sistema contemporáneo de imágenes diagnósticas con aplicaciones específicas en el área maxilofacial que permite al clínico la visualización tridimensional del área de interés, con lo que se superan las limitaciones de la radiografía convencional. Estudios indican que la TCHC tiene una mayor sensibilidad y especificidad que las RP en la detección de fracturas o fisuras; sin embargo, existe la posibilidad de falsos positivos o negativos, atribuidos principalmente a la resolución espacial del estudio en relación con la extensión y el ancho de la fractura, y a la presencia de ruido o artefactos que las simulan e impiden su visualización, generados principalmente por dientes endodónticamente tratados ^9^. Por ello, la TCHC no está específicamente indicada para esta tarea diagnóstica, pero sí cuando los signos y síntomas de un caso no son evidenciados por otros métodos o son ambiguos, y se sospecha la presencia de fractura radicular. La TCHC permite observar signos tempranos de pérdida del soporte óseo periradicular, lo que indica una fractura en la raíz adyacente al defecto ^6^^,^^8^^,^^9^^,^^11^^-^^14^. La presencia de un trazo hipodenso que se extiende en la raíz completa o parcialmente, y está presente en al menos dos cortes consecutivos, es descrita como una característica tomográfica para detectar fracturas radiculares ^15^. La posibilidad de poder visualizarlas mediante la TCHC dependerá del grado de separación de los fragmentos ^10^. 

El examen mediante TCHC debe adaptarse a las necesidades particulares del caso, lo que implica el conocimiento clínico del mismo, la evaluación de imágenes radiográficas previas y considerar que sus beneficios superen los potenciales riesgos. La optimización de los parámetros de adquisición del volumen tomográfico con fines endodónticos, así como la sistematización de la observación del mismo, permiten una valoración precisa, siendo crítica en la identificación de cambios sutiles en las estructuras duras del diente como los producidos por una fisura ^10^^,^^12^^,^^14^^,^^16^^,^^20^. Considerando lo expuesto, la presente serie de casos describe las estrategias empleadas para la adquisición de la TCHC y la exploración dinámica de las imágenes en tres casos con sospecha de fractura radicular. 

## PRESENTACIÓN DE LOS CASOS

### Adquisición de las imágenes tomográficas

Los estudios de TCHC fueron adquiridos utilizando un equipo Promax Classic 3D (Planmeca, Helsinki, Finlandia), utilizando un campo de visión (FOV-*Field of view*) de 5 x 5 cm y un tamaño de voxel entre 0,100 y 0,150 mm, empleando 90 Kv y 8-12 mA, con un tiempo de exposición de 11-14 segundos. 

### Estrategia de análisis del volumen tomográfico

**Figura 1:**
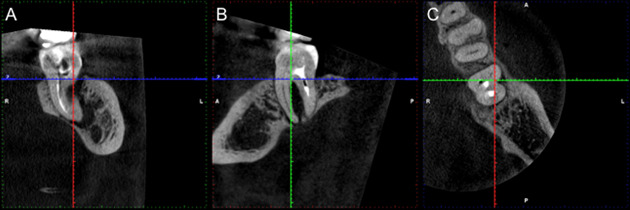
Representación del reformateo oblicuo de los planos o rotación del volumen tomográfico para establecer un paralelismo entre estos y el eje mayor del diente con el fin de evitar distorsiones. En los diferentes cortes (A: coronal; B. sagital, C: axial).

Los volúmenes tomográficos fueron analizados empleando el *software* Romexis versión 6.1 (Planmeca, Helsinki, Finlandia). La observación y navegación dinámica de las reconstrucciones multiplanares se realizó en la ventana “explorador”, mediante el reformateo oblicuo de los planos o rotando el volumen para establecer un paralelismo entre éstos y el eje mayor del diente, con el fin de evitar distorsiones. Para el recorrido del volumen se ajustó el espesor del corte y la distancia entre cortes al valor mínimo permitido por el *software*. Los parámetros de nitidez, brillo, contraste y *zoom* se emplearon con la finalidad de mejorar la visualización de las imágenes. En los diferentes cortes se analizó la presencia o ausencia de defectos óseos y líneas hipodensas; cuando estaban presentes, debían ser observados en al menos tres cortes consecutivos, para encontrar la relación entre ambos (Fig. 1).

### Caso 1

**Figura 2:**
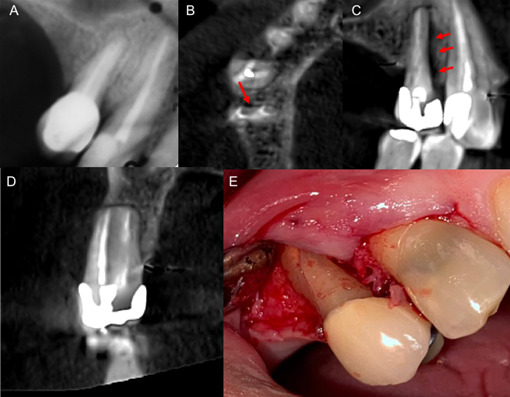
Caso 1. (A) Radiografía periapical donde se observan el ensanchamiento en el espesor del ligamento periodontal y moderado defecto óseo mesial. Cortes axial (B) y sagital (C) de tomografía computarizada de haz cónico que permiten evidenciar la amplitud del defecto óseo vertical mesial. Corte coronal (D) en el que se constata la ausencia de la tabla ósea vestibular. Fotografía clínica (E) obtenida durante la elevación del colgajo, que confirma la dehiscencia y la fractura.

Paciente masculino de 68 años, quien consulta por dolor de varios días de evolución en la región maxilar derecha. Al examen clínico se observa restauración protésica en la unidad dentaria (UD) 1.4, absceso periodontal y fístula con drenaje purulento, positivo a la percusión y palpación. Fueron evidentes la presencia de facetas de desgaste en las unidades dentarias 1.3,1.2 y 1.1 y el cambio de coloración en la UD 1.3. La RP reveló conductos aparentemente sellados, presencia de ensanchamiento del espacio del ligamento periradicular e imagen sugestiva de moderado defecto óseo vertical mesial (Fig. 2A). Se indica la adquisición de la TCHC para ampliar el estudio radiológico. Mediante el estudio del volumen tomográfico fue posible observar la restauración protésica con retención intracameral y dos conductos con sellado hiperdenso; las vistas axiales y sagitales permitieron identificar el defecto óseo vertical mesial a lo largo de la pared mesial (Figs. 2B-C); en las vistas coronales se constató la ausencia de la tabla ósea vestibular (Fig. 2D). La presencia de artefactos dificultó la identificación de alguna línea de fractura. La fotografía clínica obtenida durante la elevación del colgajo, una vez que cedieron los síntomas agudos, confirmó la fractura y fue realizada la exodoncia (Fig. 2E).

### Caso 2 

**Figura 3:**
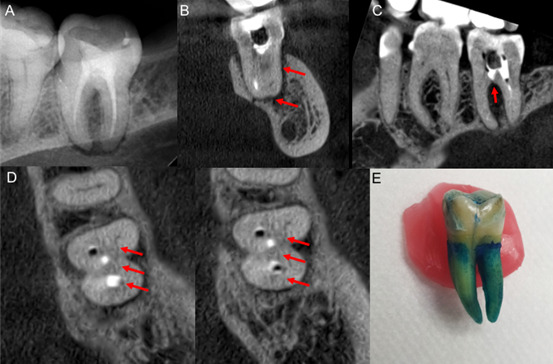
Caso 2. (A) RP permite observar la imagen radiolúcida de la lesión periapical y furca y la obturación de los conductos radiculares. Cortes coronal y sagital (B y C): el sellado hiperdenso parcial de los conductos, defecto óseo con separación de la tabla ósea vestibular y el compromiso de la región de furca. Cortes axiales (D) observación de las líneas de fractura mesiodistal y vestibular. (E) fotografïa clínica que evedencia la fractura longitudinal corono-radicular.

Paciente masculino de 63 años, quien consulta por dolor de días de evolución localizado en la UD 3.7, que se incrementa con la masticación; refiere tratamiento endodóntico de hace cuatro años. Al examen intrabucal se observó edema gingival vestibular, positivo a percusión y palpación, sondaje mayor de 5 mm en vestibular de la UD 3.7 y movilidad grado II. En la RP (Fig. 3A) se observó imagen radiolúcida periapical, se indicó el retratamiento endodóntico precediendo a la desobturación para facilitar la descongestión y el alivio de la sintomatología. Mediante la TCHC se observó en los cortes coronales y sagitales (Fig. 3B-C) sellado hiperdenso parcial de los conductos, extenso defecto óseo vestibular por la separación entre la tabla ósea vestibular y la raíz, así como el compromiso en la región de furca. Los cortes axiales (Fig. 3D) permitieron la observación de las líneas de fractura mesio-distal y vestibular. La fotografía clínica (Fig. 3E) posterior a la exodoncia puso en evidencia la fractura longitudinal corono-radicular. 

### Caso 3 

**Figura 4:**
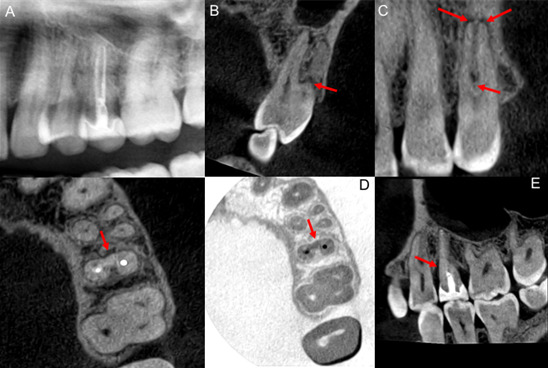
Caso 3. Detalle de radiografía panorámica donde no se evidencian signos radiológicos de fisura o lesión periapical. Corte coronal (B) y corte sagital (C) de tomografía computarizada de haz cónico en donde se evidencia imagen hipodensa en región de furca y región periapical del diente 2.4, y una línea hipodensa compatible con fractura. Cortes axiales (D) y sagital (E) que muestran el defecto óseo mesial en la unidad dentaria 2.5.

**Figura 5:**
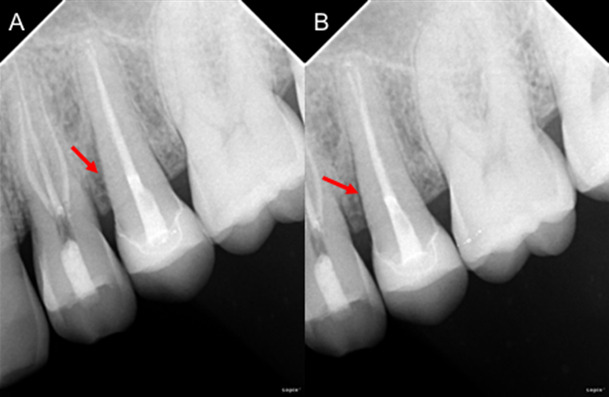
Caso 3. Radiografías periapicales postoperatorias que con variación de la angulacion horizontal que permiten evidenciar limitadamente el defecto óseo mesial al diente 2.5.

Paciente masculino de 44 años, quien refiere dolor en región premolar superior izquierda. Relata antecedente de fisura radicular vertical hace cinco años en la UD 2.5 con tratamiento endodóntico y realización de corona, encontrándose asintomático hasta el momento. Al examen intrabucal de la UD 2.4 se evidenció dolor a la percusión y palpación apical, diente sin caries o restauración, respuesta negativa al test de vitalidad pulpar y ausencia de bolsa periodontal; la UD 2.5 fue positiva a palpación y percusión, movilidad grado II y sondaje de 6 mm en mesial. La radiografía panorámica no fue conclusiva (Fig. 4A). En los cortes sagitales y coronales de TCHC (Figs. 4B-C) se observó el hallazgo de la pérdida ósea en la zona de furca y periapical de la UD 2.4; los cortes coronales permitieron la visualización de una línea hipodensa compatible con fractura. En la UD 2.5, los cortes sagitales y axiales (Figs. 4D-E) permitieron evidenciar el defecto óseo mesial a la UD 2.5 por fisura previa. Las RP (Figs. 5A-B) muestran la conducta clínica conservadora de tratar endodónticamente la UD 2.4 y expectante con la UD 2.5 al ceder los síntomas clínicos después del tratamiento. 

## DISCUSIÓN

La disrupcion longitudinal completa o incompleta del esmalte, la dentina y posiblemente el cemento son denominadas de una manera general como fracturas ^1^ y constituyen la tercera causa de pérdida de unidades dentarias después de la caries y la enfermedad periodontal ^3^. Su diagnóstico es un desafío para el clínico debido a su mal pronóstico, especialmente cuando no son detectadas a tiempo ^2^. Este diagnóstico es el resultado de una combinación del examen clínico y de los estudios imagenológicos donde la TCHC juega un rol importante ^4^^,^^7^.

Los tres casos reportados coinciden con la literatura que indica una mayor prevalencia de fracturas en dientes endodónticamente tratados ^3^^,^^4^, lo que es atribuido a que éstos reúnen generalmente el antecedente de fisuras y fracturas previas, debilitamientos de la estructura dentaria en la región pericervical, cambios en el grosor de las paredes generados por las técnicas de instrumentación y en las propiedades de la dentina producidos por las soluciones irrigadoras y medicaciones intraconducto ^6^. Asimismo, todas las fisuras fueron observadas en dientes posteriores, lo que parece estar relacionado con la topografía oclusal, presencia de itsmos, formas planas, paredes delgadas que representan puntos débiles a las cargas de fuerzas funcionales y parafuncionales ^5^^,^^6^. La edad de los pacientes estuvo entre los 30 y 69 años, como reportan estudios previos ^3^.

De acuerdo con Bueno *et al*. ^16^, las líneas hipodensas de formas curvas e irregulares son sugestivas de fracturas y pueden ser diferenciadas de los artefactos que son rectos y mínimamente irregulares. Asimismo, las líneas hipodensas que son observadas durante la navegación continua en varios cortes secuenciales pueden sugerir fracturas y se distinguen del ruido, el cual desaparece después de tres o cuatro cortes. Señalan que la presencia de material de obturación que penetra en una línea de fractura puede ser un aspecto diferenciador de un artefacto y la asociación de ésta con una reabsorción ósea. La línea de fractura es el epicentro de la agresión, lo cual provoca que la lesión ósea sea equidistante a ella, cuando existen defectos óseos verticales y la causa es una fractura; ese defecto tiende a ser más recto y menos abierto que cuando se trata de enfermedad periodontal. Por otra parte, consideran que el artefacto hipodenso debe observarse desde la fuente que lo genera y en todo su recorrido, el cual normalmente sobrepasa los límites del diente, lo que no ocurre con la línea de fractura. 

Gao *et al*. ^20^ proponen una clasificación de acuerdo con la apariencia tomográfica de la fractura en: 1. Desplazadas, 2. Sutiles y 3. Escondidas. Las fracturas reportadas pudieron ser catalogadas como sutiles en los casos 2 y 3 (UD 2.4) y escondidas en los casos 1 y 3 (UD 2.5). En éstos fue común la asociación entre la pérdida ósea detectada en la TCHC y la presencia de fractura, lo que sirvió como hallazgo indirecto para su búsqueda en la imagen y puede demostrar la presencia en fotografias clínicas. Fayad *et al*. ^7^ reportaron hallazgos similares en relación al defecto óseo vertical de los casos 1 y 3, la separación de la tabla ósea de la superficie radicular como fue evidenciado en el caso 2 y las lesiones de furca en los casos 2 y 3. Los hallazgos coinciden con el estudio de Zhang *et al*. ^11^, quienes compararon tomográficamente la posibilidad de detectar la fractura o el patrón de pérdida ósea, y encontraron que existe una mayor sensibilidad y seguridad en la detección de patrones de pérdida ósea, que sugieren la presencia de fractura y confirman la utilidad del estudio tomográfico. Esto coincide con lo indicado por Chavda *et al*. ^17^, quienes analizaron 21 dientes diagnosticados como insalvables y señalan que la TCHC es más precisa en la identificación de cambios sutiles tempranos en el hueso perirradicular que en la detección de la línea de fractura. 

La posibilidad de observar tomográficamente la línea de fractura está directamente relacionada con su ancho y la calidad de la imagen obtenida. La calidad depende de diversos factores cuyo resultado final es la resolución espacial del estudio ^10^^,^^16^^,^^17^. Durante la adquisición de los volúmenes tomográficos de los casos presentados en este reporte, fueron consideradas una serie de estrategias propuestas por Bechara *et al*^19^, Gao *et al*. ^20^, Byacova *et al*. ^10^, Bueno *et al*. ^16^, Dias *et al*. ^12^ y Oliveira *et al*. ^18^, quienes indican que en los estudios tomográficos de dientes con sospecha de fractura, deben utilizarse un tamaño de voxel menor a 0,150 mm, aumentar el kV, mA y la cantidad de imágenes base, lo que incrementa la resolución espacial, de contraste y disminuye la expresión de artefactos. Asimismo, los autores plantean que el diente de interés debe ser posicionado lo más céntrico posible en el volumen, inmovilizando al paciente para reducir los artefactos de movimiento. La utilización de un campo de visión inferior a 5 cm es clave para disminuir la dosis de radiación y la cantidad de tejidos expuestos.

La exploración de cada volumen fue realizada en sus planos axial, coronal y sagital siguiendo un orden de análisis dinámico, orientado a la inspección del defecto óseo y su asociación o no con una línea de fractura que estuviera presente en al menos tres cortes consecutivos, a un mínimo espesor e intervalo entre corte y corte, respetando el paralelismo de los ejes con la pared dentinaria y con el trazo hipodenso que, en conjunto con la evaluación clínica, permitió la decisión de la exodoncia de los casos 1 y 2 y la conducta conservadora y expectante en el caso 3. La TCHC representa también el punto de partida para la planificación de los procedimientos posteriores.

## CONCLUSIONES

El diagnóstico de fisuras y fracturas depende del engranaje de múltiples factores clínicos e imagenológicos para la toma de decisiones terapéuticas y es siempre desafiante. En los casos reportados, la TCHC aportó información relevante para considerar la exodoncia o una conducta conservadora con la preservación de la unidad dentaria. La justificación y optimización del examen tomográfico deben ser adaptadas a las necesidades particulares del caso, lo que implica el conocimiento clínico del mismo, imágenes radiográficas previas y que sus beneficios superen los potenciales riesgos de la dosis. La estrategia de una exploración dinámica y sistemática de las imágenes en los tres planos del espacio, además del conocimiento de cómo las fisuras o fracturas pueden ser identificadas, fueron elementos que posibilitaron una valoración más precisa de los casos aquí presentados.

## Fuentes de financiamiento:

Autofinanciado
